# Molecular Analysis and Modeling of Hepatitis E Virus Helicase and Identification of Novel Inhibitors by Virtual Screening

**DOI:** 10.1155/2018/5753804

**Published:** 2018-08-30

**Authors:** Mohammad K. Parvez, Naidu Subbarao

**Affiliations:** ^1^Department of Pharmacognosy, College of Pharmacy, King Saud University, Riyadh, Saudi Arabia; ^2^School of Computational and Integrative Sciences, Jawaharlal Nehru University, New Delhi, India

## Abstract

The hepatitis E virus- (HEV-) helicase as a novel drug-target was evaluated. While cell culture model was used for mutational characterization of helicase,* in silico* protein modeling and virtual screening were employed to identify helicase inhibitors. None of the saturation mutant replicons significantly affected RNA replication. Notably, mutants encompassing the Walker motifs replicated as wild-type, showing indispensability of nucleotides conservation in viability compared to known criticality of amino acids. A 3D modeling of HEV-helicase and screening of a compound dataset identified ten most promising inhibitors with drug likeness, notably, JFD02650, RDR03130, and HTS11136 that interacted with Walker A residues Gly975, Gly978, Ser979, and Gly980. Our model building and virtual identification of novel helicase inhibitors warrant further studies towards developing anti-HEV drugs.

## 1. Introduction

The hepatitis E virus (HEV) is an emerging pathogen that causes acute hepatitis E in general and chronic infection in immunocompromised individuals [1, 2]. Globally, hepatitis E accounts for about 70,000 deaths that include up to 30% of pregnant women per year [3, 4]. HEV classified within the genus* Orthohepevirus* of the family Hepeviridae has at least seven genotypes (HEV1-HEV7), of which four (HEV1-HEV4) are known to infect humans [5]. Compared to the HEV1 and HEV2 strains, HEV3 and HEV4 are less pathogenic but potentially zoonotic to swine as well as other farm and wild mammalian species [6]. The HEV single-stranded positive sense RNA genome (~7.2 kb) consists of three open reading frames (ORF1, ORF2, and ORF3) [7]. Of these, the ORF1 encodes the viral nonstructural polyprotein, essential for its life cycle [8]. Based on* in silico* polyprotein sequence analysis of genetically-close viruses, methyltransferase (MeT/MTase), Y (undefined), papain-like cysteine protease (PCP), proline-rich hinge/hypervariable region (PPR/HVR), X (undefined/macro), helicase (hel/NTPase), and RNA-dependent RNA polymerase (RdRp) domains had been proposed in HEV ORF1 [9]. Further molecular and/or biochemical characterizations of MTase [10, 11], PCP [12], HVR [13, 14], X [15, 16], RdRp [17, 18], and Y [19] domains have shown their crucial roles in virus replication and infectivity. However, despite extensive efforts on expressing ORF1, its activity as a multifunctional polyprotein or individually active proteins still remain debatable [8].

The HEV-hel/NTPase sequences (HEV1-ORF1; a.a. 960-1204) are mapped between X and RdRp domains [9]. It belongs to superfamily 1 (SF1) helicases with signature motifs (I, Ia, II, III, IV, V, and VI) and is shown to have multiple enzymatic functions [20]. SF1 helicases of positive sense RNA viruses, however, remain less well-characterized. HEV-hel contains highly conserved Walker A (a.a. 975-982) and Walker B (a.a. 1029-1032) motifs with nucleoside triphosphate (NTP) and magnesium ion (Mg^2+^) binding activity, respectively [20]. As a functional protein, when expressed in prokaryotic system, HEV-hel showed both NTPase and 5′-3′ RNA duplex unwinding activities that were abolished upon introducing mutations in the Walker motifs [21]. It has been also suggested that HEV-hel possess *γ*-phosphatase activity, probably involved in the first step of 5′ RNA capping* in vitro* [21]. In addition, HEV replicon with mutations in Walker motifs are shown to abrogate RNA replication in hepatoma cells [22]. Nevertheless, the role of helicase domain nucleotide sequences or their conservations in HEV replication remains inclusive.

Because of self-limiting acute manifestation in general population, there has been no established treatment for hepatitis E. However, with the recent emergence of chronic infections, interferon-*α* (pegIFN-*α*-2a) and ribavirin (RBV) have become the off-label drugs of choice [23–25]. Though RBV effectively clears the virus and induces sustained virological responses, emergence of HEV-RdRp mutants (e.g., G1634R) leads to drug-resistance or nonresponse in a proportion of patients [26, 27]. The HEV-hel with established activities, therefore, offers an attractive drug-target. In view of this and the available information, the present study further extends molecular analysis of the HEV-hel domain, including protein modeling and virtual screening of potential helicase inhibitors as future anti-HEV drug candidates.

## 2. Materials and Methods

### 2.1. Construction of HEV-hel Domain Mutant Replicons

**Figure 1:**
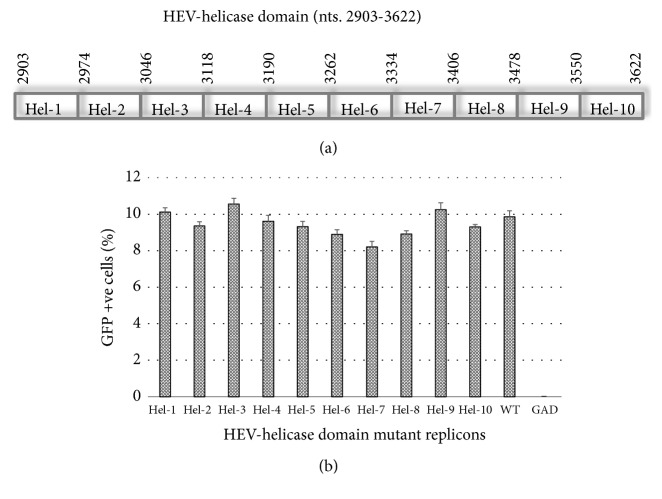
Mutational analysis of the hepatitis E virus helicase (HEV-hel) domain (nts. 2903-3622). (a) Schematic representation of the HEV-hel domain saturation mutants (Hel-1 through Hel-10; ~72 bases each). (b) Flow cytometry analysis of GFP positive S10-3 cells, showing the replication competence of mutant replicons. Hel-1:* pSK-GFP-hel1* (nts. 2903-2974); Hel-2:* pSK-GFP-hel2* (nts. 2975-3046); Hel-3:* pSK-GFP-hel3* (nts. 3047-3118); Hel-4:* pSK-GFP-hel4* (nts. 3119-3190); Hel-5:* pSK-GFP-hel5* (nts. 3191-3262); Hel-6:* pSK-GFP-hel6* (nts. 3263-3334); Hel-7:* pSK-GFP-hel7* (nts. 3335-3406); Hel-8:* pSK-GFP-hel8* (nts. 3407-3478); Hel-9:* pSK-GFP-hel9* (nts. 3479-3550); Hel-10:* pSK-GFP-hel10* (nts. 3551-3622); WT:* pSK-GFP *(positive control); and GAD:* pSK-GFP-GAD *(negative control). Data are presented as mean** ±** standard error of mean (*n*=3).

The green-fluorescence protein (GFP) reporter HEV replicons* pSK-GFP-WT *(HEV1-SAR55; GenBank no. AF444002) and* pSK-GFP-GAD *(RdRp mutant) were gifted by Dr. Suzanne Emerson (NIAID, National Institutes of Health, USA). A series of ten consecutive saturation mutants (*pSK-GFP-hel1* through* pSK-GFP-hel10*; nts. 2903-3622; ~72 bases each) that completely covered the HEV-hel domain (Figure 1(a)) were constructed by site-directed mutagenesis [12]. In brief, polymerase chain-reaction (PCR) was carried out in a 50 *μ*l reaction volume, using 10 ng of DNA (* pSK-GFP*), appropriate amounts of mutant primers, dNTPs mix, DNA polymerase, and buffer under prescribed thermal conditions (TaKaRa Bio Inc., Japan). The amplicons were* Dpn I* (Invitrogen, USA) digested, transformed (heat-shock) into DH5*α* XL-blue competent cells (Strata gene, USA), and selected on ampicillin-agar plates. Plasmids (Qiagen Plasmid Mini-prep Kit, Germany) were confirmed for induced mutations by sequencing (Invitrogen, USA) and DNA stocks (Qiagen Plasmid Maxi-prep Kit, Germany) were stored (-20°C).

### 2.2. Hepatoma Cell Culture

S10-3, the human hepatoma cell line (gift from Dr. Suzanne Emerson, NIAID, NIH, USA), was cultured in complete DMEM media as described elsewhere [28] at 37°C with 5% CO_2_ supply. S10-3 cells (0.5 x10^6^/well) were seeded in 24-well flat-bottom plates for transfection experiments.

### 2.3. In Vitro Transcription and Transfection

All replicons were* in vitro* transcribed as capped genomic RNA and transfected into S10-3 cells, essentially as described elsewhere [28]. Transfected cells were further incubated at 34.5°C for six days to allow optimal RNA replication and GFP production. The* pSK-GFP-WT *and* pSK-GFP-GAD* transfected cells served as positive and negative control, respectively. All transfections were done in duplicate and repeated twice.

### 2.4. Fluorescence Microscopy and Flow Cytometry

The transfected cells were monitored on days 2, 4, and 6 for GFP production, the indicator of RNA replication under fluorescence microscope (Nikon H600L). The transfected cells were harvested using trypsin (Invitrogen, USA) on day 6 and processed as described previously [29]. Briefly, cells were first collected in cold phosphate buffered saline (PBS; 200 *μ*l/well, each) and recollected by rinsing the well with another 200 *μ*l of PBS. Each culture (in duplicate) was pelleted at 4°C, resuspended in 300 *μ*l of cold PBS, and immediately subjected to flow cytometry (10,000 cell count/sample) and data were analyzed for GFP positive cells.

### 2.5. HEV-hel Model Building and Molecular Dynamic Simulation

The HEV-hel (HEV1-ORF1; a.a. 963-1194) was modeled (corresponding to a.a. 1-232) using Swiss-modeler [30]. By using sequence comparison and functionally conserved domain search methods, the best matched helicase structure of tomato mosaic virus (ToMV) (PDB code: 3WRX) with 33% identity was evaluated as the suitable template for modeling. The GROMAC was used to perform the molecular dynamic simulation task [31].

### 2.6. Molecular Docking

The molecular docking tools AutoDock Vina [32] and Genetic Optimization for Ligand Docking (GOLD) 5.0 [33] were used for virtual screening of the candidate compounds dataset (Maybridge database). Docking annealing parameters for van der Walls and hydrogen bonding were set to 5.0 and 2.5, respectively. The parameters used for genetic algorithm were of population size 100, selection pressure 1.2, number of operations 1,00,000, number of islands five, niche size 2, migrate 10, mutate 100, and cross-over 100.

### 2.7. Postdocking and Drug Likeness Analyses

The* X*-Score, a consensus scoring function was used in order to carry out docking validation [34]. It uses the negative logarithm of the dissociation constant of the ligand to the protein (−log Kd) as the average of three scoring functions (HPScore, HMScore, and HSScore). The docked complexes were analyzed for the interacting residues, using Discovery Studio software (Accelrys Discovery Studio Visualizer 2.5.5.9350). All the selected HEV-hel inhibitory compounds were further analyzed for their drug likeness parameters such as molecular weight (MW), partition coefficient (LogP), H-bond donor (HBD), H-bond acceptor (HBA), rotatable bonds (RB), and rule of 5 (Ro5).

## 3. Results

### 3.1. The HEV-hel Domain Nucleotides are Indispensable for RNA Replication

The saturation mutations, i.e., introducing each and every possible nucleotide change while conserving the amino acid residues in the replicon (cDNA) did not affect the gross yield of* in vitro* synthesized RNA (data not shown). When transfected into S10-3 cells, none of the mutant transcripts significantly affected RNA replication (Figure 1(b)). However,* pSK-GFP-hel7* mutant (nts. 3335-3406) had a mild downregulation of replication as compared to the wild-type (*pSK-GFP-WT*). Notably, the* pSK-GFP-hel1* (nts. 2903-2974) and* pSK-GFP-hel3 *(nts. 3047-3118) mutants encompassing the Walker A and Walker B motifs replicated close to the wild-type. In addition, our replicon* pSK-GFP-hel4* (nts. 3119-3190) with the mutant codons of naturally detected L1110 and V1120 substitution also had no effect on RNA replication (Figure 1(b)). This very clearly showed the nonconservation and indispensability of HEV-hel nucleotide sequences on virus replication at transcriptional level. Notably, the reported mutational studies on recombinant HEV-hel protein as well as replicon have established the enzymatic functions of helicase domain and its essentiality in RNA replication [21, 22, 35, 36]. Taken together, our mutational analysis further endorsed the enzymatic functions of HEV-hel domain.

### 3.2. Validation of the Model

**Figure 2:**
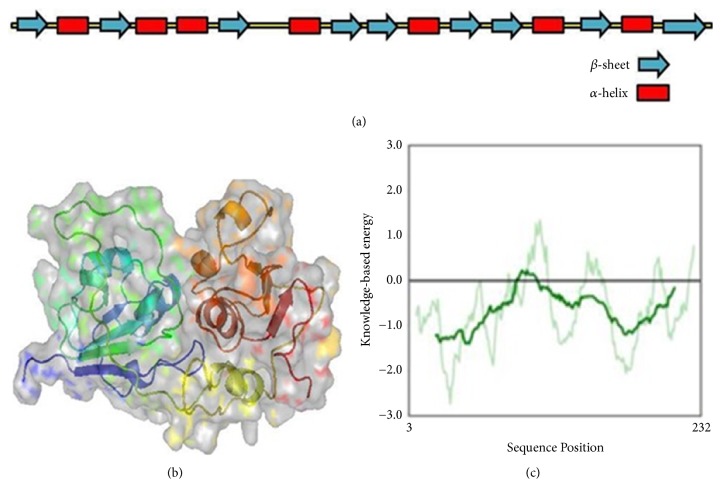
The hepatitis E virus helicase (HEV-hel) model. (a) The secondary structures within the HEV-hel (HEV1-ORF1; a.a. 963-1194) model. (b) The deduced 3D structure of HEV-hel. (c) The Prosa validation plot.

The ToMV-helicase with best matched identity (33%) served as the suitable template for HEV-hel modeling. The modeled HEV-hel protein (residues no. 1-232 corresponding to HEV1 a.a. 963-1194) was validated by drawing Ramachandran plot (https://www.ebi.ac.uk/thornton-srv/software/PROCHECK/). It showed that while the phi-psi angles of 85.8% of the HEV-hel residues were in favored regions, 11.2% were in additionally allowed regions, 0.5% were in generously allowed regions, and 2.5% in the disallowed regions. The helicase model was also checked in Prosaweb server [37]. The verified 3D analysis showed that the deduced model had an averaged Z score of -6.4. All results indicated that the HEV-hel protein model was valid (Figure 2).

### 3.3. Molecular Dynamics Simulation

**Figure 3:**
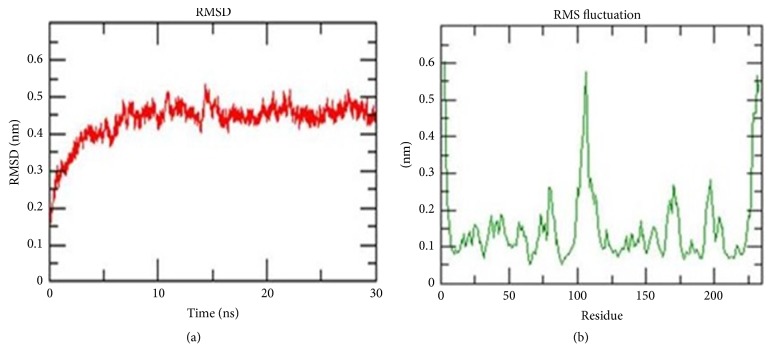
Molecular dynamic simulation results. (a) The root mean square deviation (RMSD) plot, showing the change of protein backbone (nanometer) against simulation time (nanosecond). (b) The root mean square fluctuation (RMSF) plot, reflecting the mobility of a certain residue around its mean position (nanometer).

The stability and properties of the structure of the homolog were studied by explicit solvent molecular dynamic (MD) simulation. The root mean square deviation (RMSD) analysis not only reflects the change of protein backbone versus simulation time but also indicates the divergence of two structures. The RMSD of homolog became stable at 10 ns. The RMSD value of the modeled HEV-helicase was 0.4 nm (Figure 3). This result indicated that an accepted structure was obtained by the simulation and it was reliable for further analyses. The root mean square fluctuation (RMSF) reflects the mobility of a certain residue around its mean position, which is another tool for studying the dynamics stability of the system. Although there are some deviations among the trajectories (especially in loop region), the present data suggested less fluctuations which further highlighted the reliability of the modeled structure (Figure 3).

### 3.4. Molecular Docking and Drug Likeness

**Figure 4:**
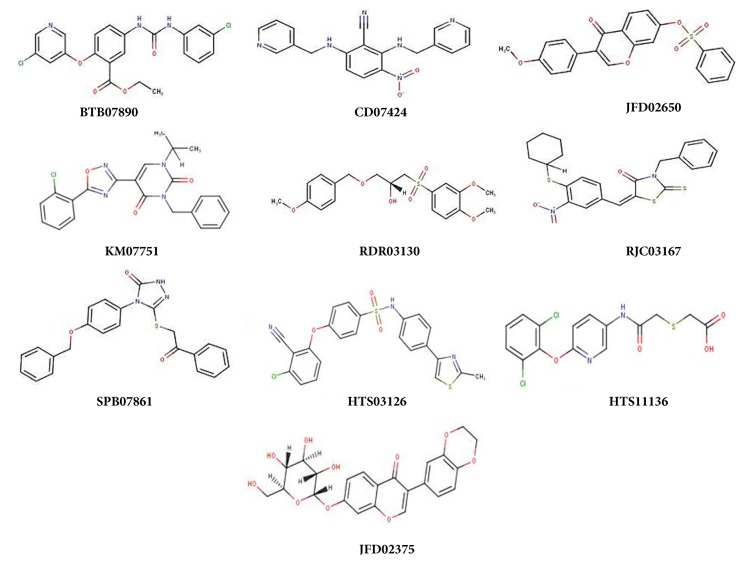
The 2D structures of selected inhibitory compounds of HEV-helicase (source: Maybridge database).

**Figure 5:**
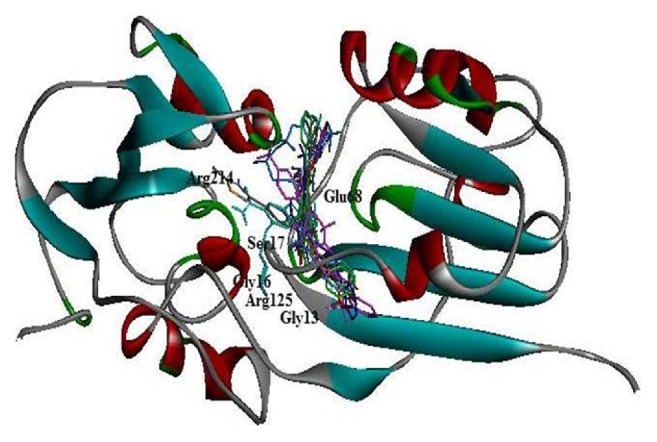
Binding interaction of the best selected compounds with the HEV-helicase active residues. The modeled protein (residues no. 1-232 corresponding to HEV-hel a.a. 963-1194) shows the interacting residues Gly13 (HEV-hel Gly975), Gly16 ((HEV-hel Gly978), Ser17 (HEV-hel Ser979), and Gly18 (HEV-hel Gly980) within the highly conserved Walker A motif.

To identify the best inhibitor molecules, a database of ~14000 compounds (selected from 56000 compounds dataset) were docked in the active site of HEV-hel using the AutoDock Vina and GOLD docking programs. The docked compounds were ranked based on their highest binding energy and GOLD Fitness Score with the corresponding protein. Finally, the top 10 ranked hits with higher binding and GOLD Fitness Score were considered and investigated further for their mode of interaction with the crucial residues (no. 1-232 corresponding to HEV a.a. 963-1194) in the modeled protein. The selected compounds (Figure 4) with their virtual binding mechanisms are summarized (Table 1 and Figure 5). All compounds showed high binding energies ranging from −6.9 to −8.9 kcal/mol with AutoDock Vina and GOLD fitness Score ranging from 80 to 87 (Table 1). Moreover, these compounds also had reasonable binding energies as predicted by X-Score (Table 1). It was found that residues Gly16, Ser17, Gly18, Arg125, and Arg214 formed significant interactions with the inhibitor compounds (Figure 5). Interestingly, while Gly16, Ser17, and Gly18 in the modeled protein corresponded to the HEV-hel Walker A motif residues Gly978, Ser979, and Gly980, these residues were hydrogen bonded with all the selected compounds. In addition, these compounds also stabilized the complex through the hydrophobic and other nonbonded interactions. Notably, the highest hydrogen bonds and hydrophobic contacts were formed by compounds JFD02650 and RDR03130 followed by HTS11136 which made at least 4 to 5 hydrogen bonds with Gly13, Gly16, Ser17, Gly91, Arg125, Thr185, and Arg214 residues of the modeled HEV-hel. Notably, these three compounds also interacted with the Gly13 corresponding to Walker A residue Gly975 in addition to Gly978, Ser979, and Gly980. Moreover, all the selected compounds also showed drug likeness properties except RJC03167 that violated one property of rule of 5 (Table 2).

## 4. Discussions

In the current age of chronic HEV infections [2], pegIFN-*α*-2a and RBV have become the antiviral drugs of choice [23–25]. However, RBV leads to the emergence of viral RdRp-mutants accountable for drug nonresponse or failure in a proportion of chronic patients [26, 27]. The HEV-hel with established activities, therefore, offers an attractive target towards developing new and efficacious anti-HEV drugs. In this study, we analyzed the effects of HEV-hel domain sequential nucleotide mutations on RNA replication using* in vitro* replicon-cell culture model, followed by* in silico* protein modeling, molecular simulation, helicase-inhibitors screening, and drug likeness studies. Of a series of ten mutant replicons tested, none significantly affected RNA replication, showing the indispensability of HEV-hel nucleotides on virus replication. In contrast, the HEV RNA replication was abolished upon introducing amino acid mutations in both Walker A and Walker B motifs [22]. Moreover, the recombinant HEV-hel protein with mutations within the Walker motifs when expressed in* E. coli* lost its enzymatic activities* in vitro* [21]. Notably, the frequently detected natural HEV-hel L1110F and V1120I substitutions (downstream of Walker B) in fulminant liver failure patients [35] are shown to decrease the protein's ATPase activity as well as RNA replication in cultured cells [22]. Contrarily, our replicon containing mutations in the corresponding codons of L1110 and V1120 also did not affect the RNA replication. In another* in vitro* study, deletions in the HEV-hel Ia and III motifs significantly impaired ATPase and unwinding activities [36]. Taken together, our saturation mutation analysis further endorsed the enzymatic function of HEV-hel domain.

Because HEV1 and HEV2 are very pathogenic compared to HEV3 and HEV4 [6], the HEV1 helicase sequences were used to model the target protein. The ToMV-helicase is the only reported crystal structure of a helicase of positive sense RNA viruses [38]. Therefore, the ToMV-helicase with best matched identity served as the suitable template to produce a validated 3D structure of HEV-hel, for the first time. Notably, a previous prediction of the HEV-hel 3D structure (Phyre 2 server) also used ToMV helicase that, however, only showed the presence of alpha helices (%) and beta sheets (%) [36]. Our further studies on stability and properties of the structure by explicit solvent molecular dynamic simulation parameters (RMSD and RMSF) indicated its acceptability and reliability. Molecular docking of a database of compounds in the active site of modeled HEV-hel led to the top 10 ranked hits with higher binding mechanisms. Of these, the highest hydrogen bonds and hydrophobic contacts were formed by JFD02650, RDR03130, and HTS11136 molecules. Interestingly, these compounds strongly interacted with the highly conserved Walker A motif residues Gly975, Gly978, Ser979, and Gly980 that were shown critical for the HEV-hel enzyme activities [20]. Moreover, all the selected helicase-binding compounds also showed drug likeness properties except RJC03167.

## 5. Conclusion

Our saturation mutation analysis along with the helicase protein expression data strongly endorses the enzymatic function of HEV-hel domain. Moreover, the modeled 3D structure of HEV-hel enables virtual identification of ten most promising helicase inhibitors, notably, JFD02650, RDR03130, and HTS11136 with drug likeness properties that warrant further studies towards developing novel anti-HEV drugs.

## Figures and Tables

**Table 1:** Molecular docking parameters of the selected compounds binding affinity with the modeled HEV-helicase.

No.	Compounds (PubChem ID)	AD Vina (Kcal/mol)	GOLD Score	X-Score (Kcal/mol)	Hydrogen bonds	Range (Å)
1	BTB07890	-7.3	86.09	-9.40	Gly16, Ser17, Gly18	1.7-2.25
2	JFD02650	-8.9	80.15	-9.05	Gly13, Gly16, Ser17, Gly91, Arg125	2-2.4
3	CDO7424	-6.9	87.33	-8.77	Gly16, Ser17, Gly18	1.7-3.1
4	SPB07861	-8.0	83.24	-9.08	Gly13, Gly16, Gly91, Arg214	1.3-2.2
5	HTS03126	-8.8	80.63	-8.56	Gly16, Ser17, Arg125	1.2
6	RDR03130	-7.7	85.27	-7.06	Gly16, Ser17, Arg125, Thr185, Arg214	1.4-2.8
7	HTS11136	-7.0	82.08	-8.34	Gly16, Ser17, Gly18, Gly91, Arg214	1.5-2.4
8	JFD02375	-8.2	83.29	-8.45	Gly16, Ser17, Glu68, Arg214	1.6-3.1
9	KM07751	-8.3	80.57	-9.44	Gly16, Ser17, Arg214	1.2-1.9
10	RJC03167	-7.6	81.82	-8.70	Gly16, Ser17, Arg125	1.8-2.2

**Table 2:** Drug likeness parameters of the selected compounds showing HEV-helicase inhibitory activities.

No.	Compounds (PubChem ID)	MW	HBD	HBA	LogP	RB	Ro5 violation
1	BTB07890	446.288	2	7	3.9	7	0
2	JFD02650	408.428	0	6	3.68	5	0
3	CD07424	360.375	2	8	2.14	6	0
4	SPB07861	417.487	1	6	3.72	8	0
5	HTS03126	481.982	1	6	3.74	6	0
6	RDR03130	396.458	1	7	1.69	8	0
7	HTS11136	387.24	2	6	3.31	7	0
8	JFD02375	458.417	4	10	0.46	4	0
9	KM07751	422.87	0	7	3.84	5	0
10	RJC03167	470.636	0	7	6.1	6	1

## Data Availability

The data (biological and computational) used to support the findings of this study are included within the article.
